# A high-density Diversity Arrays Technology (DArT) microarray for genome-wide genotyping in *Eucalyptus*

**DOI:** 10.1186/1746-4811-6-16

**Published:** 2010-06-30

**Authors:** Carolina P Sansaloni, César D Petroli, Jason Carling, Corey J Hudson, Dorothy A Steane, Alexander A Myburg, Dario Grattapaglia, René E Vaillancourt, Andrzej Kilian

**Affiliations:** 1 Plant Genetics Laboratory, EMBRAPA Genetic Resources and Biotechnology - EPqB, 70770-910 Brasilia, Brazil; 2 Dep. Cell Biology, Universidade de Brasilia - 70910-900 Brasília - DF, Brazil; 3 Diversity Arrays Technology Pty Ltd, 1 Wilf Crane Crescent, Yarralumla, ACT 2600, Australia; 4 School of Plant Science and Cooperative Research Centre for Forestry, University of Tasmania, Private Bag 55, Hobart, Tasmania 7001, Australia; 5 Department of Genetics, Forestry and Agricultural Biotechnology Institute (FABI), University of Pretoria, Pretoria, 0002, South Africa; 6 Genomic Sciences Program - Universidade Católica de Brasília - SGAN, 916 modulo B, 70790-160 Brasília - DF, Brazil

## Abstract

**Background:**

A number of molecular marker technologies have allowed important advances in the understanding of the genetics and evolution of *Eucalyptus*, a genus that includes over 700 species, some of which are used worldwide in plantation forestry. Nevertheless, the average marker density achieved with current technologies remains at the level of a few hundred markers per population. Furthermore, the transferability of markers produced with most existing technology across species and pedigrees is usually very limited. High throughput, combined with wide genome coverage and high transferability are necessary to increase the resolution, speed and utility of molecular marker technology in eucalypts. We report the development of a high-density DArT genome profiling resource and demonstrate its potential for genome-wide diversity analysis and linkage mapping in several species of *Eucalyptus*.

**Findings:**

After testing several genome complexity reduction methods we identified the *Pst*I/*Taq*I method as the most effective for *Eucalyptus *and developed 18 genomic libraries from *Pst*I/*Taq*I representations of 64 different *Eucalyptus *species. A total of 23,808 cloned DNA fragments were screened and 13,300 (56%) were found to be polymorphic among 284 individuals. After a redundancy analysis, 6,528 markers were selected for the operational array and these were supplemented with 1,152 additional clones taken from a library made from the *E. grandis *tree whose genome has been sequenced. Performance validation for diversity studies revealed 4,752 polymorphic markers among 174 individuals. Additionally, 5,013 markers showed segregation when screened using six inter-specific mapping pedigrees, with an average of 2,211 polymorphic markers per pedigree and a minimum of 859 polymorphic markers that were shared between any two pedigrees.

**Conclusions:**

This operational DArT array will deliver 1,000-2,000 polymorphic markers for linkage mapping in most eucalypt pedigrees and thus provide high genome coverage. This array will also provide a high-throughput platform for population genetics and phylogenetics in *Eucalyptus*. The transferability of DArT across species and pedigrees is particularly valuable for a large genus such as *Eucalyptus *and will facilitate the transfer of information between different studies. Furthermore, the DArT marker array will provide a high-resolution link between phenotypes in populations and the *Eucalyptus *reference genome, which will soon be completed.

## Background

A number of molecular marker technologies have been developed and used for species of *Eucalyptus *in the last 20 years [1]. Each of these technologies allowed important advances in the understanding of the multifaceted genetics, evolution and breeding of this vast genus that includes over 700 species, some of which are globally important plantation forestry species [2]. Molecular markers have been used to resolve phylogenetic issues [3], describe the genetic structure of natural populations [4,5], solve questions related to the management of genetic variation in breeding populations [6] and build linkage maps [7-9] that in turn have led to the identification of QTLs for important traits [10-13]. Nevertheless, the genotyping density achieved even with technologies such as AFLP [14] remains at a few hundred markers per sample and because AFLP is gel-based it is relatively labour-intensive. Multiplexing has allowed moderate-level throughput in microsatellite studies. However, the transferability of microsatellites across species is notoriously poor and needs to be investigated and optimized before microsatellites can be used in a new species [1]. Wider genome coverage and higher throughput genotyping methods are necessary to increase resolution and speed for a variety of applications. Diversity Arrays Technology (DArT) [15] provides a promising alternative to satisfy the requirements of throughput, genome coverage and transferability. DArT is a complexity reduction, DNA hybridization-based method that simultaneously assays hundreds to thousands of markers across a genome. DArT preferentially targets low-copy genomic regions, allows automation of data acquisition and is cost competitive. Although developed some years ago, this marker technology has recently gained increasing attention [16-20]. We report the development of the first version of a high density operational DArT genotyping microarray with over 7,000 markers and demonstrate its potential for diversity and linkage mapping studies in species of *Eucalyptus *across the two most important subgenera.

## Results and Discussion

**Figure 1:**
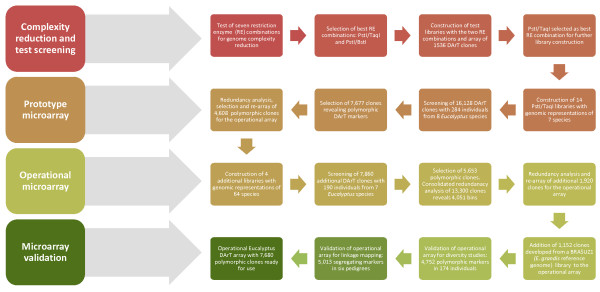
**Flowchart of the development of the DArT genotyping microarray for *Eucalyptus***.

This paper describes the various steps that were taken in developing the eucalypt DArT array (Figure 1). The first step was to find a successful method for reducing genome complexity. Once this was done, a prototype microarray was developed and tested. The DArT array was subsequently expanded and again tested for redundancy. The final step was to validate the operational microarray for genome-wide genotyping in *Eucalyptus*.

### Genome complexity reduction

The first necessary step in the development of DArT markers (Figure 1) is choosing a genome complexity reduction method (see http://www.diversityarrays.com/molecularprincip.html). The DArT genome complexity reduction method is based on restriction enzyme (RE) digestion of total genomic DNA, adapter ligation and amplification of adapter-ligated fragments. DNA extraction was done with a CTAB protocol [21]. Seven methods of genome complexity reduction were tested for their performance in *Eucalyptus *(Additional File 1). DNA samples were prepared by digestion with the rare cutting *Pst*I RE as a primary cutter in combination with a frequently cutting enzyme (*Taq*I, *Bst*NI, *Msp*I, *Hpa*II, *Ban*II, *Mse*I or *Alu*I) as a secondary cutter. *Pst*I is sensitive to CpG methylation, thereby excluding heavily methylated repetitive DNA from the representation. Adapters, complementary to the "sticky-ends" of the fragments generated by *Pst*I digests were ligated (protocol modified slightly from the original [15,16]), to allow PCR amplification of only the *Pst*I fragments that had not been cut with the secondary enzyme. A desirable genome complexity reduction method will produce a smear of products with few or no distinct bands when representations are visualised on agarose gels following electrophoresis. Strong banding indicates the amplification of repetitive sequences and such representations are unsuitable for DArT development [22]. The genomic representations produced by the digestion with *Pst*I in combination with either *Taq*I (*Pst*I*/Taq*I) or *Bst*NI (*Pst*I/*Bst*NI) were considered the most suitable for *Eucalyptus *to advance to the subsequent development steps (Figure 1, Additional File 1).

### Test screening of clones for polymorphic DArT markers

The second step (Figure 1) entailed the construction of small genomic libraries for each of the selected complexity reduction methods and the screening of the resulting DNA clones (probes) to reveal polymorphic markers. For library construction, two sets of pooled DNA samples were utilized separately: the first from 12 *E. grandis *and the second from 12 *E. globulus *trees. Each pooled sample was digested with both enzyme combinations: *Pst*I/*Taq*I and *Pst*I/*Bst*NI. Four testing libraries were generated, each with 384 randomly picked clones, with a total of 1,536 DArT clones to be screened for polymorphism. The cloned DNA fragments were printed onto glass slides for the first test array in duplicates (randomly positioned within the array) as is normally done for DArT. Genomic representations of each of the 12 *E. grandis *and 12 *E. globulus *individuals were prepared to generate 'targets' that were hybridized to the arrays. For each species, the 12 genotypes were assayed with two technical replicates per genotype. Each target was labeled with a green fluorescent dye (Cy3-dUTP) and red fluorescent dye (Cy5-dUTP), and then mixed with a blue fluorescently-labeled polylinker from the vector used for cloning the DNA fragments in the libraries that provided a reference value for the quantity of amplified DNA fragment present in each 'spot' of the microarray, as well as an in-built quality control for spots on the microarrays. This mixture was hybridized to a 1,536-clone microarray, that was scanned for blue, green & red fluorescence and data were extracted using *DArTSoft *version 7.44. *DArTSoft *localizes the individual spot features of the microarrays and then compares the relative intensity (blue versus green) and (blue versus red) values obtained for each clone across all slides/targets to detect the presence of clusters of higher and lower values corresponding to marker scores of '1' (high) and '0' (low) respectively. The quality parameters used in this study were: Call Rate (percentage of targets that could be scored as '0' or '1') and Reproducibility value (reproducibility of scoring between replicated target assays) [16]. The results of the *DArTSoft *analysis for the two arrays prepared using DNA clones derived from either *Pst*I/*Taq*I or *Pst*I/*Bst*NI digestions were compared with regard to the frequency of clones revealing polymorphic DArT markers. The criteria used for declaring a clone as revealing a polymorphic marker were Reproducibility > 97% and Call Rate > 80%. From the analysis of the two species hybridized in duplicate to the two arrays, the complexity reduction method using *Pst*I/*Taq*I was found to yield a higher proportion (21.7%) of candidate polymorphic markers according to the above criteria compared to the *Pst*I/*Bst*NI method (14.3%).

### *Prototype *Eucalyptus *DArT microarray*

The *Pst*I/*Taq*I genome complexity reduction method was used in the development of the prototype *Eucalyptus *DArT microarray (Figure 1). The initial test array, with 1,536 clones, was expanded by picking an additional random set of 14,592 discovery DArT clones, this time derived from a total of 14 libraries (Table 1). A broader sample of genotypes (254 samples from seven eucalypt species representing the two most important genera of eucalypts [*Corymbia *and *Eucalyptus*] and the two most important subgenera of *Eucalyptus *[*Eucalyptus *and *Symphyomyrtus*]) were used for library construction resulting in a broader sample of DNA sequences, therefore increasing the probability of sampling genomic segments that could reveal polymorphic markers across a wider range of genetic backgrounds [16]. A total of 16,128 clones were printed twice on each slide and were hybridized with DNA from each of 284 individuals ("targets"; Table 2) representing eight different species with replication, following the methods described above. The results were analyzed with *DArTSoft *and assessed using the threshold criteria of Reproducibility > 97% and Call Rate > 80%. This analysis revealed 7,677 clones (47.6%) as robust polymorphic markers (Table 3). The Call Rate average was 95.3% and the Reproducibility average was 99.7% (this value was calculated on the basis of duplicate genotyping assays for all test samples).

**Table 1:** Libraries and corresponding numbers of clones screened for the prototype *Eucalyptus *DArT microarray

No. of clones	No. of individuals	Species*	Source**
768	12	*Corymbia variegata*	Australia
768	11	*E. camaldulensis*	Australia
768	13	*E. globulus*	Portugal/Chile
1920	12	*E. globulus*	Australia
1536	24	*E. globulus*	Australia
768	12	*E. globulus*	Australia
1536	96	*E. grandis × E. urophylla*	Brazil
1536	12	*E. grandis*	South Africa
2688	9	*E. grandis*	South Africa
768	6	*E. nitens*	Chile
768	11	*E. nitens*	Australia
768	12	*E. pilularis*	Australia
768	12	*E. urophylla*	South Africa
768	12	*E. urophylla*	South Africa

* Corymbia variegata belongs to a genus phylogenetically closely related to *Eucalyptus*; *E. camaldulensis, E. globulus, E. grandis, E. urophylla *and *E. nitens *belong to *Eucalyptus *subgenus *Symphyomyrtus*; *E. pilularis *belongs to *Eucalyptus *subgenus *Eucalyptus*.

** Sourced from native stands or first-generation breeding populations established from seed stocks derived from native stands.

**Table 2:** *Eucalyptus *species and number of individuals of each species (total of 284) used as targets to screen the prototype DArT microarray for polymorphic markers

No. of individuals	Species
135	*E. grandis × E. urophylla*
28	*E. pilularis*
27	*E. nitens*
35	*E. globulus*
12	*E. cladocalyx*
12	*E. grandis*
12	*E. urophylla*
12	*Corymbia variegata*
11	*E. camaldulensis*

**Table 3:** Summary of results of the *Eucalyptus *DArT microarray development involving screening for polymorphism and score signature-based redundancy analysis in the prototype and operational arrays (see text for details; n.d. not determined)

Technology development phase	No. of DArT clones screened	No. and (%) of polymorphic DArT clones	No. of bins with unique scoring pattern
Initial libraries	16,128	7,677 (47.6%)	2,652 (16.4%)
Array expansion libraries	7,680	5,653 (73.6%)	n.d.
All libraries	23,808	13,300 (55.9%)	4,051 (17.0%)

Testing *Corymbia *targets on the array composed primarily of *Eucalyptus *probes (and *vice versa*) showed very clearly that the overall array signal of *Corymbia *targets was low and uncorrelated to signal from *Eucalyptus *species (and *vice versa*). Because of this poor transferability across genera, we abandoned the development of DArT for *Corymbia*. As clones used to build an array are picked at random from the libraries, clone redundancy (i.e. DNA fragments with the same or very similar/overlapping sequence) is an issue. Redundancy of the polymorphic DArT clones was evaluated with the software package *DArT ToolBox *http://www.diversityarrays.com/ by constructing a Hamming distance matrix between clones, followed by distance binning, in which all clones with zero distance were placed into the same bin. This was done using the 284 samples used as targets listed in Table 2. This estimation of clone redundancy based on similar score pattern enabled the selection of unique or low redundancy clones prior to the availability of sequence information for the clones. The redundancy estimation based on distance binning of the 7,677 polymorphic markers resulted in 2,652 unique bins, i.e. 34.5% non-redundant marker scoring patterns (Table 4). With a limited number of effective scores for calculating the distance matrix for markers and a clear genetic structure in the materials used for initial marker discovery, there was a high likelihood of unique sequences being grouped to a single bin, especially in large bins. Therefore, a total of 4,608 clones were selected for re-arraying, keeping approximately 30% of the potentially redundant markers, with frequency of retention proportional to the bin size. In order to verify the redundancy estimation, we sequenced re-arrayed clones that belonged to nine bins that had at least 30 clones. Sequencing results revealed that on average 53% of the DArT clones in these large bins represented unique DNA sequences. Binning results were therefore, as anticipated, conservative and yielded an overestimation of redundancy (Table 5).

**Table 4:** Distribution of the number of polymorphic DArT clones within each binning class in the prototype and interim phases of the DArT microarray development

No. of clones in bin	No. of bins in prototype array *(7,677 polymorphic clones)*	No. of bins in interim array *(13,300 polymorphic clones)*
1	1,330	2,143
2-9	1,199	1,737
10-19	105	126
20-29	9	17
30-39	4	8
40-49	2	3
≥ 50	3	17
Total	2,652	4,051

**Table 5:** Results of DArT clone redundancy analysis based on DNA sequencing of clones selected from the nine bins that had at least 30 clones per bin, based on Hamming distance of zero (no difference in scoring pattern between markers in the bin)

Bin #	No. of clones per bin based on Hamming distance	No. of clones selected for re-arraying and sequencing	No. of re-arrayed clones with unique DNA sequences	% of re-arrayed clones with unique DNA sequences
1	116	33	19	57.6
2	75	20	12	60
3	59	17	8	47.1
4	43	13	6	46.2
5	41	6	4	66.7
6	39	10	5	50
7	37	16	11	68.8
8	31	10	6	60
9	30	9	2	22.2
Average	52.3	14.8	8.1	53.2

### Interim and operational Eucalyptus DArT microarrays

In order to enrich the *Eucalyptus *DArT array for polymorphic markers, four additional genomic libraries were constructed that provided a total of 7,680 new clones that were screened for polymorphism (Table 6). Two of these libraries contained DNA from 62 eucalypt species and were built by pooling equimolar DNA quantities from one individual of each species and cutting either with *Pst*I or *Pst*I/*Taq*I. The *Pst*I representation allowed markers that were present at low frequency in the *Pst*I/*Taq*I representation to be cloned and therefore minimized redundancy in the final clone set. Most species (56) were from subgenus *Symphyomyrtus *(representing 14 of the 15 sections and missing only a minor one); the other species were from three other subgenera (*Alveolata, Eucalyptus*, and *Minutifructus*). Screening these new libraries for polymorphism (Figure 1) was performed using a set of 190 individuals from seven different *Eucalyptus *species (*E. grandis, E. urophylla, E. camaldulensis, E. globulus, E. dunni, E. pilularis and E. nitens*) with targets in full replication (Table 7). *DArTSoft *and *DArT ToolBox *were used to identify robust markers and to estimate redundancy as described for the first array (with the same parameters and thresholds). *DArTSoft *detected 5,653 polymorphic markers among the 7,680 clones (73.6%). The average Call Rate and Reproducibility were similar to the first array with 93.7% and 99.7% respectively. However, a significantly higher percentage of polymorphic markers (73.6% versus 47.6%) was found in the array expansion stage (Table 3), most likely due to the greater genetic diversity that was captured in the genomic representations from the four new libraries. A consolidated analysis of redundancy based on binning was carried out to minimize redundancy between the 7,677 polymorphic clones selected initially for the prototype microarray and the additional 5,653 clones. From a total of 13,300 clones, 4,051 bins were found in the interim array (Tables 3 and 4). On the basis of polymorphism analysis and the additional redundancy assessment, 1,920 new clones were selected from the 7,680, to create a second re-arrayed library. The two re-arrayed libraries (the first one with 4,608 clones and the second with 1,920 clones), were supplemented with 1,152 clones developed primarily from a genomic library of BRASUZ1, the *Eucalyptus grandis *tree whose genome is being sequenced (Table 6), to constitute an operational DArT genotyping array for *Eucalyptus *with 7,680 markers.

**Table 6:** Four additional genomic representation libraries and corresponding numbers of clones used for the development of the interim and operational DArT microarray

No. of clones in library	No. of individuals	Species	Source	RE digestion
1920 *	16	*E. grandis × E. urophylla (IP pedigree)*	Brazil	*Pst*I/*Taq*I
	16	*E. grandis × E. urophylla (VCP pedigree)*	Brazil	*Pst*I/*Taq*I
	16	*E. camaldulensis × (E. urophylla × E. globulus)*	Brazil	*Pst*I/*Taq*I
	16	*(E. grandis × E. urophylla) × (E. urophylla × E. globulus)*	Brazil	*Pst*I/*Taq*I
	16	*(E. dunnii × E. grandis) × (E. urophylla × E. globulus)*	Brazil	*Pst*I/*Taq*I
	16	*(E. dunnii × E. grandis) × E. urophylla*	Brazil	*Pst*I/*Taq*I
1152 **	1	*E. grandis (BRASUZ1)*	Brazil	*Pst*I/*Taq*I
2304 ***	62	Several species	Australia	*Pst*I/*Taq*I
2304 ***	62	Several species	Australia	*Pst*I

* Library built by pooling equimolar quantities of DNA of 96 inter-specific hybrids.

** Library built with DNA of *E. grandis *tree BRASUZ1, whose full genome is being sequenced.

*** The following species were used: *E. albens, E. balladoniensis, E. bicostata, E. biterranea, E. brassiana, E. brevistylis, E. camaldulensis, E. cladocalyx, E. coolabah, E. cordata, E. cornuta, E. cosmophylla, E. crebra, E. dalrympleana, E. deglupta, E. delicata, E. diversicolor, E. dundasii, E. dunnii, E. falcata, E. glaucescens, E. glaucina, E. globulus, E. gomphocephala, E. grandis, E. gunnii, E. hallii, E. houseana, E. howittiana, E. leucophloia, E. lockyeri, E. longifolia, E. lucasii, E. maidenii, E. michaeliana, E. microcorys, E. morrisbyi, E. nitens, E. obtusiflora, E. optima, E. ovata, E. pachycalyx, E. pachyphylla, E. paludicola, E. perriniana, E. platyphylla, E. polyanthemos, E. populnea, E. pseudoglobulus, E. pulverulenta, E. pumila, E. ravereana, E. rubida, E. salmonophloia, E. scoparia, E. stoatei, E. tereticornis, E. torquata, E. urophylla, E. viminalis, E. wandoo, E. woodwardii*.

**Table 7:** *Eucalyptus *pedigrees and corresponding numbers of individuals used as targets to screen the 7,680 clones for degree of polymorphism

No. of individuals	Species
71	*E. grandis × E. urophylla*
16	*E. camaldulensis × (E. urophylla × E. globulus)*
16	*(E. grandis × E. urophylla) × (E. urophylla × E. globulus)*
16	*(E. dunnii × E. grandis) × (E. urophylla × E. globulus)*
16	*(E. dunnii × E. grandis) × E. urophylla*
16	*E. pilularis*
16	*E. nitens*
23	*E. globulus*

### Validation of DArT array for diversity and linkage mapping

The performance of the operational DArT array for diversity studies was first validated by genotyping 174 individuals from six of the *Eucalyptus *species used to create the libraries (*E. grandis, E. urophylla, E. dunni, E. camaldulensis, E. globulus *and *E. nitens*). These individuals were a subset of those used to create the libraries. This analysis revealed 4,752 polymorphic markers out of the 7,680 clones (61.9%) among the 174 individuals. As expected, not all the 7,680 clones were found to yield polymorphic markers since the 174 samples assayed did not represent the total genetic diversity used to construct the array.

As a second validation, an assessment of DArT marker segregation and rate of polymorphism was carried-out with 94 samples in full replication, including 15-16 samples from each of six mapping pedigrees. Most of these individuals were not used in library construction and represented a test of the level of polymorphism that could be expected in diverse linkage mapping experiments. There were 2,211 polymorphic markers per pedigree on average (Table 8). The number of shared polymorphic markers (polymorphic in two pedigrees) amongst the six mapping pedigrees varied from a minimum of 859 to a maximum of 1,328 (Table 8). A total of 5,013 markers (65.3%) out of the 7,680 clones showed segregation within at least one mapping population, when data from the six pedigrees were consolidated (Table 9). Table 9 shows the number of DArT markers that were exclusively polymorphic in one pedigree only (1,154 markers or 23%) through to those that were polymorphic in an increasing number of pedigrees up to all six pedigrees (150 markers: 3%).

**Table 8:** Number of polymorphic DArT markers in each *Eucalyptus *mapping pedigree (diagonal) and shared among mapping pedigrees (above the diagonal)

	C1 × UGl	DG × U	DG × UGl	G × U(IP)	UGl × GU	G × U(VCP)
C1 × UGl	2394	864	1328	1123	1172	899
DG × U		1818	1154	1029	866	859
DG × UGl			2465	1251	1284	953
G × U(IP)				2553	1175	1144
UGl × GU					2176	946
G × U(VCP)						1861

C1 × UGl = *E. camaldulensis *× (*E. urophylla × E. globulus*); DG × U = (*E. dunnii × E. grandis*) × *E. urophylla*; DG × UGl = (*E. dunnii × E. grandis*) × (*E. urophylla × E. globulus*); G × U(IP) = *E. grandis *× *E. urophylla *(pedigree IP); UGlxGU = (*E. urophylla × E. globulus*) × (*E.grandis × E. urophylla*); G × U(VCP) = *E. grandis × E. urophylla *(VCP pedigree).

**Table 9:** Informativeness of DArT markers from the operational array for genetic mapping based on sampling six different pedigrees (see Table 8 for list of pedigrees)

No. of mapping pedigrees in which a marker was polymorphic	No. of DArT markers in the class	% of total number of polymorphic markers
1	1,407	28.1
2	1,154	23.0
3	1,048	21.0
4	761	15.2
5	493	9.8
6	150	3.0
Total	5,013	

## Conclusions

This eucalypt DArT array is one of the best performing DArT arrays yet developed (DArT Pty Ltd, unpublished results). The high frequency of polymorphic markers is likely to be a function of the high level of sequence variation in the *Eucalyptus *genome [23] and, to a much lesser extent, a function of its relatively small genome size and low proportion of repetitive DNA [1]. Interestingly, the high level of sequence diversity in *Eucalyptus *species [23] could be a serious impediment to the development of highly multiplexed SNP platforms that usually require reasonably long stretches of sequence without secondary SNPs. It may prove challenging to find good targets for SNP assay design which would be invariable across a range of *Eucalyptus *species. In this context, DArT analysis is not constrained by high sequence polymorphism and is therefore very suitable for genotyping thousands of genetic markers in highly outbred organisms such as *Eucalyptus*.

DArT generated a substantially larger number of robust polymorphic markers for *Eucalyptus *species than previous technologies. Although co-dominant microsatellites are significantly more informative at the single locus level they are low-throughput and expensive per data-point. Comparing DArT with RAPD or AFLP analysis would be more appropriate as they are all dominant markers. The complicating issue, however, is the ascertainment bias that takes place when selecting RAPD primers, AFLP primer/enzyme combinations or DArT polymorphic probes. This bias is exacerbated by the specific target population that is used when selecting polymorphisms and by the rigor of the experimenter in declaring these polymorphisms. It is important to note that the DArT array developed in this study provides at least two orders of magnitude more polymorphic markers in a single assay than RAPD or AFLP analysis. In *Eucalyptus*, while a selected RAPD primer can provide up to 10 robust polymorphic bands in a single gel run and a selected AFLP combination can provide on average 30 to 40 polymorphic markers, a single DArT assay provides 1,000 to 4,000 polymorphic markers from the7,680 probes present on the current array. In addition, the standard probe set selected for routine DArT genotyping allows comparisons of markers across a range of species and populations while both AFLP and RAPD markers are much less amenable to integration across laboratories and even less so across different species.

The high level of DArT marker multiplexing was validated in a large collection of eucalypt species and individuals. The results indicated that the DArT genotyping array will deliver thousands of polymorphic markers for population diversity studies and provide a very efficient platform with which to generate high-density linkage maps with a substantial proportion of markers shared across pedigrees. This array will be especially useful for applications that benefit from access to a large number of markers. The cost per data point (per sample per marker) will of course depend on the application and the facility generating the data. Using the fully costed service provided by the technology development partner, DArT Pty Ltd, the cost per data point for polymorphic markers is expected to vary between one and five cents US (assuming an assay cost per sample of 50 USD, not counting shipping and DNA extraction costs). In linkage mapping studies, an application where one of the lowest degrees of polymorphism is expected because diversity comes essentially from only two parents, we expect that a minimum of 1,000 polymorphic markers could be mapped at a cost of approximately five cents US per polymorphic marker. The per sample cost is much cheaper than current SNP genotyping platforms assaying an equivalent number of markers (e.g. Illumina GoldenGate). The in-house use of DArT arrays would involve purchasing the equipment necessary to spot high density arrays, hybridization chambers and a multi color scanner and therefore would require a very high throughput operation to make such investment worthwhile.

Another significant advantage of the DArT markers is their transferability across species, which is particularly valuable when dealing with a genus like *Eucalyptus *with over 700 species, of which many are foundation species in their forest ecosystems, and several are commercially useful in either temperate or sub-tropical regions of the world. This transferability will allow the detailed comparison of linkage maps and QTL positions across studies. However, this transferability appears to have limits as we obtained poor transferability across eucalypt genera (*Corymbia *to *Eucalyptus*). We will address the phylogenetic consequences of this finding and the performance of the DArT array across the full range of *Eucalyptus *species in a related study (Steane *et al. *submitted).

A limitation of the DArT technology compared to multi allelic microsatellites is their dominant inheritance, which precludes studying aspects of within-individual variation, although methodologies are being developed that can mitigate this [24]. Dominant markers are also less informative for constructing linkage maps, unless a large number of them are available and population sizes are large, in which case they can be as useful as co-dominant markers. Finally, clustering of DArT markers across the genome could potentially be an issue due to the reduced representation method by which that DArT probes are developed. However, this is not exclusive to the DArT technology and an assessment of this will only be possible by linkage mapping DArT markers in multiple pedigrees and/or physically mapping them on the upcoming *Eucalyptus *reference genome.

To better characterize the genomic content of this array, all 7,680 DNA clones on the operational DArT array are being sequenced. The availability of DNA sequences for the DArT markers will facilitate the integration of high-density maps and QTL locations with the *Eucalyptus *genome assembly. The operational DArT array constitutes a powerful tool with which to undertake high resolution genetic analyses required for applications such as fine QTL mapping, genome-wide selection and complex phylogenetic and evolutionary investigations. Moreover, the flexibility and expandability of the DArT technology opens the possibility of further enriching the current array with additional polymorphic markers by simply screening additional sets of clones. A number of mapping (Grattapaglia *et al. *in prep; Kullan *et al. *in prep), population and phylogenetic (Steane *et al. *submitted) studies currently underway with DArT in several *Eucalyptus *species are corroborating the excellent performance of this technology and will be the subject of upcoming reports.

## Methods

For the development of the *Eucalyptus *DArT microarray, DNA samples from many different species and provenances were used both in the prototype and technology development steps (Tables 1, 2, 6 and 7). DNA was extracted from either fresh leaf tissue or bark cambium in three different laboratories (Australia, South Africa, Brazil) all using a CTAB protocol [21]. DNA quality was checked on agarose gels with DNA digested with the restriction enzyme *Hind*III together with undigested DNA to check that (1) undigested DNA formed a tight band of high molecular weight without RNA contamination; (2) fully-digested DNA formed a smear of mid- to low molecular weight. DNA concentration was adjusted to 50-100 ng/μL, targeting a concentration of 75 ng/μL.

### Methods of genome complexity reduction to generate genomic representations

Digestion and adapter ligation were performed simultaneously on 75 ng of genomic DNA in a 10 μL aqueous solution containing 2 Units of each restriction enzyme, 80 Units of T4 DNA Ligase and 0.05 μM adapter (5'-CAC GAT GGA TCC AGT GCA-3' annealed with 5'-CTG GAT CCA TCG TGC A-3'). Reactions were incubated at 37°C for 2 hours, followed by 2 hours at 60°C as required by the enzyme combinations. 1 μL of digestion/ligation reaction product was used as a template for PCR amplification in a 50 μL reaction using DArT *Pst*I primer (5'-GAT GGA TCC AGT GCA G-3') with the following cycling parameters: 94°C for 1 min, followed by 30 cycles of 94°C for 20 sec, 58°C for 40 sec, 72°C for 1 min, and finished with an extension at 72°C for 7 min. Initial assessment of the tested methods was performed by resolving 5 μL of amplification product in a 1.2% agarose gel stained with ethidium bromide.

### Construction of small clone DArT libraries

The genomic representations of each species/complexity reduction method combination were pooled and cloned using the *TOPO TA Cloning Kit *(Invitrogen) as specified by the manufacturer's instructions. Individual bacterial colonies were picked into 384-well plates containing LB medium with 4.4% glycerol, 100 μg/mL ampicillin and a mixture of salts to facilitate PCR from the LB cultures (unpublished observation) and grown at 37° for 18 hours. A PCR amplification was performed using 0.5 μL of bacterial culture template, 0.2 μM "M13 Forward" and "M13 Reverse" primers (Invitrogen), and the following PCR program: 95° for 4 min, 57° for 35 sec, 72° for 1 min, followed by 35 cycles of 94° for 35 sec, 52° for 35 sec and 72° for 1 min and a final step of 72° for 7 min. The PCR products were dried at 37°C and washed with 70% ethanol before being dissolved in "DArTspotter" spotting buffer, designed for use with poly-L-lysine coated micro-array slides (Wenzl *et. al. *in preparation, available from DArT Pty Ltd). Arrays were spotted using a *MicrogridII *arrayer (Biorobotics) on poly-L-lysine coated glass microarray slides (Erie Scientific). Slides were aged on the bench for 24 hours before being immersed in Milli-Q water at 95°C for 2 min, to denature the DNA spotted onto the slides, then in Milli-Q water with 0.1 mM DTT and 0.1 mM EDTA at 20°C, and finally being dried by centrifugation at 500 × g for 7 min and vacuum desiccation for 30 min.

### Fluorescent labeling of genomic representations

Genomic representations of the 12 samples of *E. grandis *and *E. globulus *were prepared as described above for library construction, to generate 'targets' for hybridizing to the arrays. The products of amplification were precipitated individually with isopropanol, washed with 70% ethanol and air dried at room temperature for 12 hours. For each species the 12 genotypes were assayed with two replicates per genotype. Targets were labeled in a 10 μL reaction volume with 2.5 nM of Cy3-dUTP or Cy5-dUTP (Amersham Bioscience), 2.5 units of Klenow exo^- ^fragment of *E. coli *Polymerase I (New England Biolabs) and 25 μM random decamers in 1 × NEB Buffer 2 (New England Biolabs). The labelling reactions were incubated at 37°C for 3 hours.

### Test hybridization to microarrays

The labeled targets were mixed with a hybridisation buffer containing a 50:5:1 mixture of Express Hyb (Clonetech), herring sperm DNA (Promega) and FAM-labeled polylinker region of the pCR 2.1 TOPO vector (Invitrogen) used for cloning the libraries, plus 2 mM EDTA at pH 8.0. The target mixtures were denatured at 95°C for 2 min before hybridization to the microarrays, which was carried out at 62.5°C for 18 hours. After hybridization, the microarray slides were washed in four solutions of increasing stringency (1 × SSC, 0.1% SDS for 4 min; 1 × SSC for 4 min; 0.2 × SSC for 1 min; 0.02 × SSC for 30 sec) and dried by centrifugation at 500 × g for 7 min and vacuum desiccation for 30 min.

### Microarray imaging and data extraction

Microarrays were scanned using a TECAN LS300 confocal laser microarray scanner at a resolution of 20 μm per pixel with sequential acquisition of 3 images for each microarray slide, using the following laser/emission-filter combinations: 488 nm laser/520 nm filter (for imaging the fluorescent signal from the FAM-labeled polylinker region of the pCR 2.1 TOPO vector); 543 nm laser/590 nm filter (for imaging the fluorescent signal from the hybridized target labeled with Cy-3); 633 nm laser/670 nm filter (for imaging the fluorescent signal from the hybridized target labeled with Cy-5). The use of a third fluorescent dye is not absolutely required and DArT assays can be performed on any two-color scanner as reported in early DArT papers. However, the third dye provides significantly higher sample throughput together with lower assay cost because two samples can be processed on a single array instead of just one as is the case when using a two-color scanner. The signal from the FAM-labeled vector polylinker provided a reference value for quantity of amplified DNA fragment present in each 'spot' of the microarray. The resulting images were analyzed using *DArTSoft *version 7.44, a program created by *Diversity Arrays Technology Pty Ltd *for microarray image data extraction, polymorphism detection, and marker scoring (Cayla *et al*. in preparation). *DArTsoft *localized the individual spot features of the microarrays from the 16 bit TIFF images generated by the laser scanner and spots with insufficient or absent reference signals were rejected from further analysis. A relative hybridisation intensity value was then calculated for all accepted spots as log [Cy-3 signal/FAM signal] for the targets labelled with Cy-3, and log [Cy-5 signal/FAM signal] for targets labelled with Cy-5. *DArTSoft *then compared the relative intensity values obtained for each clone across all slides/targets to detect the presence of clusters of higher and lower values corresponding to marker scores of '1' and '0' respectively. Targets with relative intensity values that could not be assigned to either of the clusters were recorded as unscored. For each clone, the software generated a range of quality parameters to assist in selection of polymorphic clones. The quality parameters used in this study were: Call Rate (percentage of targets that could be scored as '0' or '1') and a Reproducibility value (reproducibility of scoring between replicated target assays). Two replicates per clone were spotted on each array. The operational array has 15,360 spots in total, comprising two randomly positioned spots for each one of the 7,680 clones. The DArT array is available to the public through Diversity Arrays Technology Pty Ltd http://www.diversityarrays.com/.

## Competing interests

JC and AK are employees of Diversity Arrays Technology Pty Ltd which offers genome profiling service with the product of this report and therefore can potentially benefit from this work.

## Authors' contributions

CPS, CDP, DAS and JC performed the laboratory work, and most data analysis and interpretation; DAS and CJH participated in initial library constructions; AK, DAS, REV and AAM contributed to the design of the study; AK supervised the study, participated in data analysis and interpretation and edited the manuscript. CPS, CDP and JC drafted the initial version of the manuscript; DG and REV substantially edited the manuscript and participated in data interpretation, analysis and organization. All authors read, edited and approved the final manuscript.

## Supplementary Material

Additional file 1 **Genome complexity reduction with seven restriction enzymes**. Results of the seven restriction enzyme combinations tested for genome complexity reduction in *Eucalyptus grandis *and *Eucalyptus globulus*. **Top panel**: Gel photo showing the digestion of the same pooled DNA sample of *E. grandis *and *E. globulus *with different restriction enzyme combinations: 2-3 *Pst*I(*Taq*I), 4-5 *Pst*I(*Bst*NI), 6-7 *Pst*I(*Msp*I), 8-9 *Pst*I(*Hpa*II), 10-11 *Pst*I(*Ban*II), 12-13 *Pst*I(*Mse*I), 14-15 *Pst*I(*Alu*I). **Bottom panel**: Fluorescence intensity profile of the digested *E. grandis *DNA obtained with each complexity reduction method. The molecular sizing standard (100 bp ladder) is showed in red; track 2 (dark blue *Pst*I/*Taq*I) with a smoother profile was selected as the best complexity reduction method. Click here for file

## References

[B1] Grattapaglia D Kirst M *Eucalyptus *applied genomics: from gene sequences to breeding tools New Phytologist 2008 6 911 929 10.1111/j.1469-8137.2008.02503.x 18537893

[B2] Myburg AA Potts BM Marques CM Kirst M Gion JM Grattapaglia D Grima-Pettenati J C K Eucalyptus Genome mapping and molecular breeding in plants 2007 6 Forest trees. New York, NY, USA: Springer 115 160 full_text

[B3] Steane DA Nicolle D Vaillancourt RE Potts BM Higher-level relationships among the eucalypts are resolved by ITS-sequence data Australian Systematic Botany 2002 6 49 62 10.1071/SB00039

[B4] Steane D Conod N Jones R Vaillancourt R Potts B A comparative analysis of population structure of a forest tree, *Eucalyptus globulus *(Myrtaceae), using microsatellite markers and quantitative traits Tree Genetics & Genomes 2006 6 30 38

[B5] Payn KG Dvorak WS Janse BJH Myburg AA Microsatellite diversity and genetic structure of the commercially important tropical tree species *Eucalyptus urophylla*, endemic to seven islands in eastern Indonesia Tree Genetics & Genomes 2008 6 519 530

[B6] Grattapaglia D Ribeiro VJ Rezende GD Retrospective selection of elite parent trees using paternity testing with microsatellite markers: an alternative short term breeding tactic for *Eucalyptus* Theor Appl Genet 2004 6 192 199 10.1007/s00122-004-1617-9 15004676

[B7] Byrne M Murrell JC Allen B Moran GF An integrated genetic linkage map for eucalypts using RFLP, RAPD and isozyme markers Theoretical and Applied Genetics 1995 6 869 875 10.1007/BF00223894 24169971

[B8] Brondani R Williams E Brondani C Grattapaglia D A microsatellite-based consensus linkage map for species of *Eucalyptus *and a novel set of 230 microsatellite markers for the genus BMC Plant Biology 2006 6 20 10.1186/1471-2229-6-20 16995939 PMC1599733

[B9] Thamarus K Groom K Murrell J Byrne M Moran G A genetic linkage map for *Eucalyptus globulus *with candidate loci for wood, fibre and floral traits Theor Appl Genet 2002 6 379 387 10.1007/s001220100717 12582710

[B10] Grattapaglia D Bertolucci FL Penchel R Sederoff RR Genetic mapping of quantitative trait loci controlling growth and wood quality traits in *Eucalyptus grandis *using a maternal half-sib family and RAPD markers Genetics 1996 6 1205 1214 8913761 10.1093/genetics/144.3.1205 PMC1207612

[B11] Freeman JS Whittock SP Potts BM Vaillancourt RE QTL influencing growth and wood properties in *Eucalyptus globulus* Tree Genetics & Genomes 2009 6 713 722

[B12] Junghans DT Alfenas AC Brommonschenkel SH Oda S Mello EJ Grattapaglia D Resistance to rust ( *Puccinia psidii *Winter) in *Eucalyptus*: mode of inheritance and mapping of a major gene with RAPD markers Theor Appl Genet 2003 6 175 180 10.1007/s00122-003-1415-9 14504745

[B13] Thamarus K Groom K Bradley A Raymond CA Schimleck LR Williams ER Moran GF Identification of quantitative trait loci for wood and fibre properties in two full-sib properties of *Eucalyptus globulus* Theor Appl Genet 2004 6 856 864 10.1007/s00122-004-1699-4 15133606

[B14] Myburg AA Griffin AR Sederoff RR Whetten RW Comparative genetic linkage maps of *Eucalyptus grandis*, *Eucalyptus globulus *and their F_1 _hybrid based on a double pseudo-backcross mapping approach Theor Appl Genet 2003 6 1028 1042 10.1007/s00122-003-1347-4 12838392

[B15] Jaccoud D Peng K Feinstein D Kilian A Diversity arrays: a solid state technology for sequence information independent genotyping Nucleic Acids Res 2001 6 4 E25 10.1093/nar/29.4.e25 11160945 PMC29632

[B16] Wenzl P Carling J Kudrna D Jaccoud D Huttner E Kleinhofs A Kilian A Diversity Arrays Technology (DArT) for whole-genome profiling of barley Proc Natl Acad Sci USA 2004 6 9915 9920 10.1073/pnas.0401076101 15192146 PMC470773

[B17] Wittenberg A Lee T Cayla C Kilian A Visser R Schouten H Validation of the high-throughput marker technology DArT using the model plant *Arabidopsis thaliana* Molecular Genetics and Genomics 2005 6 30 39 10.1007/s00438-005-1145-6 15937704

[B18] Akbari M Wenzl P Caig V Carling J Xia L Yang S Uszynski G Mohler V Lehmensiek A Kuchel H Diversity arrays technology (DArT) for high-throughput profiling of the hexaploid wheat genome TAG Theoretical and Applied Genetics 2006 6 1409 1420 10.1007/s00122-006-0365-4 17033786

[B19] Xia L Peng K Yang S Wenzl P Carmen de Vicente M Fregene M Kilian A DArT for high-throughput genotyping of cassava (*Manihot esculenta*) and its wild relatives TAG Theoretical and Applied Genetics 2005 6 1092 1098 10.1007/s00122-005-1937-4 15742202

[B20] Tinker NA Kilian A Wight CP Heller-Uszynska K Wenzl P Rines HW Bjornstad A Howarth CJ Jannink JL Anderson JM New DArT markers for oat provide enhanced map coverage and global germplasm characterization BMC Genomics 2009 6 39 10.1186/1471-2164-10-39 19159465 PMC2661094

[B21] Doyle JJ Doyle JL Isolation of plant DNA from fresh tissue Focus 6 13 15

[B22] Kilian A Huttner E Wenzl P Jaccoud D Carling J Caig V Evers M Heller-Uszynska K Cayla C Patarapuwadol S The fast and the cheap: SNP and DArT-based whole genome profiling for crop improvement International Congress In the Wake of the Double Helix: From the Green Revolution to the Gene Revolution: May 27-31 2003 2005 6 Bologna, Italy: Avenue Media 443 461

[B23] Suat Hui Yeoh Maintz J Foley WJ Moran GF Comparative SNP diversity among four *Eucalyptus *species for genes from secondary metabolite biosynthetic pathways BMC Genomics 2009 6 452 10.1186/1471-2164-10-452 19775472 PMC2760585

[B24] Vekemans X AFLP-SURV version 1.0. Laboratoire de Genetique et Ecologie Vegetale 2002 University Libre de Bruxelles, Belgium

