# Supplementary material for: A high-density Diversity Arrays Technology (DArT) microarray for genome-wide genotyping in Eucalyptus
Source: Plant Methods. 2010 Jun 30;6:16. doi: 10.1186/1746-4811-6-16 (PMC2903579; doi:10.1186/1746-4811-6-16)
Supplement: Additional file 1 — Genome complexity reduction with seven restriction enzymes. Results of the seven restriction enzyme combinations tested for genome complexity reduction in Eucalyptus grandis and Eucalyptus globulus. Top panel: Gel photo showing the digestion of the same pooled DNA sample of E. grandis and E. globulus with different restriction enzyme combinations: 2-3 PstI(TaqI), 4-5 PstI(BstNI), 6-7 PstI(MspI), 8-9 PstI(HpaII), 10-11 PstI(BanII), 12-13 PstI(MseI), 14-15 PstI(AluI). Bottom panel: Fluorescence intensity profile of the digested E. grandis DNA obtained with each complexity reduction method. The molecular sizing standard (100 bp ladder) is showed in red; track 2 (dark blue PstI/TaqI) with a smoother profile was selected as the best complexity reduction method. [file 1746-4811-6-16-S1.DOC]

**Additional file 1 -** Genome complexity reduction with seven restriction enzymes
